# Progress towards World Health Organization HIV Infection 95-95-95 measures in Newfoundland and Labrador, Canada

**DOI:** 10.1371/journal.pone.0305898

**Published:** 2024-06-27

**Authors:** Kayla A. Holder, Erin Ding, Diana Kao, Mostafa Shokoohi, Jason Trigg, Robert S. Hogg, Jatin Morkar, Michael D. Grant, Deborah V. Kelly

**Affiliations:** 1 Division of BioMedical Sciences, Faculty of Medicine, Memorial University of Newfoundland, St. John’s, NL, Canada; 2 British Columbia Centre for Excellence in HIV/AIDS, Vancouver, BC, Canada; 3 Dalla Lana School of Public Health, University of Toronto, Toronto, ON, Canada; 4 Faculty of Health Sciences, Simon Fraser University, Burnaby, BC, Canada; 5 Eastern Health, St. John’s, NL, Canada; 6 Memorial University of Newfoundland School of Pharmacy, St. John’s, NL, Canada; University of Ottawa, CANADA

## Abstract

The HIV program in Newfoundland and Labrador (NL) provides care for all persons living with HIV (PLWH) in NL, yet progress toward UNAIDS 95-95-95 goals for diagnosis, linkage to care and viral suppression has not previously been documented. This analysis describes engagement in HIV care and virologic outcomes for the NL cohort in 2016 and 2019 and compares this data to the Canadian HIV Observational Cohort (CANOC). A retrospective review of the NL clinic included adults aged >18 years and descriptive statistics for demographics, risk factors, and clinical variables were assessed and compared using χ^2^ test or Fisher’s Exact test (categorical) or Wilcoxon Sum Rank test (continuous). Engagement in care and virologic outcomes for the NL cohort were consistently high over the 2016 to 2019 period with > 98% on antiretroviral therapy (ART), and > 96% having a suppressed virus load. Engagement in care and virologic outcomes among PLWH in NL is high and compares favorably to a national cohort.

## Introduction

Despite tremendous improvements to combination antiretroviral therapies (cART), human immunodeficiency virus (HIV) infection continues to be a major public health issue. The World Health Organization (WHO) estimates approximately 39 million persons were living with HIV (PLWH) worldwide at the end of 2022 with 76% having access to cART [1]. In 2014, the Joint United Nations Programme on HIV/AIDS (UNAIDS) launched the ‘90-90-90’ initiative to reduce the HIV pandemic to low-occurring endemic by 2030 [2]. The goal for this campaign was to bring widespread testing and cART treatment to the vast majority of persons living with HIV (PLWH). For the 90-90-90 goal to be met, at least 73% of all PLWH worldwide would be virally suppressed by 2020. Central to achieving and maintaining a suppressed viral load (VL), PLWH must know their status, be linked with and retained in HIV clinical care and have stable access to and maintain adherence to cART [3]. In December 2020, UNAIDS introduced an accelerated plan of action to include new global targets of ‘95-95-95’ by 2025 [4]. Early knowledge of infection and treatment initiation reduces linked HIV transmission and increases endemic control in communities through increased viral suppression [5].

Although initial testing and timely cART administration are important factors in limiting HIV spread, patient linkage and retention in quality HIV care is equally vital to the success of reaching 95-95-95 goals by 2025 [4, 6–8]. The HIV care continuum necessitates dynamic engagement and navigation of steps in a cascade in which support starts with positive diagnoses and ultimately leads to viral suppression [3]. It is a series of stages that one progresses through from the time of diagnosis to successful treatment and has since become the standard by which public health, clinicians and epidemiologists monitor the success of HIV care facilities. The five main areas in the HIV care continuum include: (1) HIV diagnosis, (2) access and linkage to HIV care, (3) retention in HIV care, (4) treatment (prescribed cART), and (5) viral suppression [3, 6, 9]. The middle steps in the care continuum, access to high quality clinical support and early timing of cART initiation, are vital factors contributing to optimal health outcomes [5, 10–12].

The Provincial HIV Clinic in St. John’s, Newfoundland and Labrador (NL) provides care for all PLWH in NL. Before this study, there were no published analyses aimed at quantifying the effectiveness of HIV care in the NL HIV Clinic towards reaching the UNAIDS 95-95-95 targets. To assess our progress in achieving this goal and the quality of outpatient care provided by the clinic, we present a descriptive analysis of all PLWH receiving care through the NL Provincial HIV Program for the 2016 and 2019 calendar years. Further, data captured from NL HIV Clinic and the Canadian HIV Observational Cohort (CANOC) collaboration for 2016 were contrasted to evaluate how the standard of care within the NL HIV Clinic related to the interprovincial collaborative cohort of PLWH in participating Canadian clinics.

## Methods

### Study approval

The study was approved by the Health Research Ethics Authority of Newfoundland and Labrador Canada (HREB #2015.118), in accordance with the Declaration of Helsinki.

### Study design and setting

We conducted a retrospective analysis of routine clinical data collected regarding care provided for PLWH through the NL Provincial HIV program. This was a retrospective use of data collected for the NL HIV cohort obtained from Eastern Health medical records (NL HIV Clinic Database populated by Meditech). The NL HIV Clinic database was accessed by KAH on Dec 4, 2019 and October 29, 2020. Detailed participant records were accessed by KAH between December 4, 2019 and April 21, 2020 and by DVK between October 3–5, 2023. KAH, MDG and DVK had access to information that could identify individual participants during or after data collection. Each participant was allocated a study ID used for linking data and data was fully anonymized after access but prior to research use. The HREB did not require patient consent as this study was deemed a secondary use of data.

For the NL cohort, adults aged > 18 years with known HIV infection and at least one CD4^+^ T cell or VL measurement, or one cART refill in the calendar year of study were considered active in care and included in this analysis. Participants were considered on cART if they had undetectable viral loads or if a prescription for cART was filled. The CANOC collaboration is a longitudinal cohort of PLWH receiving antiretroviral therapy across five Canadian provinces including British Columbia, Saskatchewan, Ontario, Quebec and Newfoundland and Labrador [13, 14]. The inclusion criteria for CANOC were adults aged > 18 years with known HIV infection and antiretroviral-naïve as of January 1, 2000, but who subsequently received cART. CANOC participants with at least one CD4 or viral load measurement between January 1, 2010 and December 31, 2016, to confirm receipt of care, were considered active in care if at least one CD4^+^ T cell or viral load measurement was taken between January 1, 2016 and December 31, 2016. For this analysis, data on participants who received care from the NL clinic were removed from the CANOC group. Demographics, risk factors, and clinical variables were added to models as explanatory variables.

### Cascade definitions for the care continuum

The concept of an HIV treatment cascade is to provide a means of identifying gaps along the continuum of HIV care, which includes prevention, diagnosis and treatment [15]. As the NL HIV program provides care to all patients living with HIV throughout the province, for this study, we assumed that everyone who is diagnosed as living with HIV has been linked to care and is included in our NL cohort. Retention in care rate (cascade 3) refers to the number of individuals with at least one CD4^+^ T cell or viral load measurement for a calendar year divided by participants meant to receive care. The proportion of participants on cART (cascade 4) were individuals with at least one cART prescription in the calendar year of study divided by the number of participants retained in care. Viral suppression (cascade 5) includes participants with cART prescribed and last measure of RNA level for the year < 200 copies/mL divided by all participants on cART.

### Statistical analysis

Descriptive statistics were performed for all characteristics of the NL cohort by year, and between NL and the rest of CANOC for 2016. Data were reported as frequencies and proportions for categorical variables and median and interquartile range for continuous variables. Comparisons were made between the 2016 NL HIV Cohort and the 2016 CANOC cohort and between the 2016 NL HIV Cohort and the 2019 NL HIV Cohort. P-values of categorical variables were calculated using a χ^2^ test or Fisher’s Exact test, while p-values for continuous variables were calculated using Wilcoxon Rank Sum test. Odds ratio (OR), 95% confidence interval (CI) and *P*-values were calculated by logistic regression modelling probability of being in the 2016 NL HIV Cohort compared to being in the 2016 CANOC cohort. Statistical analyses were conducted by the BC Centre of Excellence using SAS v9.4 (SAS Institute, North Carolina, USA). Analysis comparing NL Cohort CD4^+^ T cell data was performed using GraphPad Prism v10.2.1.

## Results

### Clinic profile of the 2019 NL cohort

There were 188 participants active in care in the 2019 NL cohort. The median age was 53 years (interquartile range (IQR): 46–57 years) and participants were more likely to be male (76%) than female or transgender (24%) (Table 1). Most participants (n = 165) were born in Canada and the dominant reported HIV risk factor was unprotected anal intercourse among men having sex with men (MSM; 53%), followed by heterosexual (38%), blood, endemic or vertical transmission (13%) and intravenous (IV) drug use (7%) (Table 1). Co-infection with hepatitis C virus (HCV) or hepatitis B virus (HBV) was low (10%), and 82% were seropositive for human cytomegalovirus (HCMV) (Table 1).

**Table 1:** Characteristics of NL cohort in 2016 and 2019 and comparison with CANOC.

Characteristic	Category	Descriptive Statistics					
		NL 2016		NL 2019		CANOC 2016	
		n = 177		n = 188		n = 11768	
		n	(%)	n	(%)	n	(%)
**Age (years)**	Median (IQR)	50 (44–55)		53 (46–57)		47 (38–54)^b^	
**Gender**	Male	137	(77)	143	(76)	9744	(83)
	Female	40	(23)	44	(23)	1963	(17)
	Transgender	0	(0)	1	(1)	55	(0)
	Unknown	0	(0)	0	(0)	6	(0)
**Born In Canada**	No	12	(7)	17	(9)	1512	(13)
	Yes	163	(92)	165	(88)	2513	(21)
	Unknown	2	(1)	6	(3)	7743	(66)
**HIV Risk MSM**		91	(51)	100	(53)	5669	(48)
**HIV Risk Heterosexual**		78	(44)	71	(38)	2538	(22)
**HIV Risk IDU**		19	(11)	14	(7)	2478	(21)
**HIV Risk Blood products**		4	(2)	5	(3)	177	(2)
**HIV Risk Endemic country**		11	(6)	16	(9)	726	(6)
**HIV Risk Perinatal transmission**		1	(1)	2	(1)	8	(0)
**Age of Starting cART (years)**	Median (IQR)	37 (31–45)		37 (31–46)		39 (32–47)^b^	
**Baseline CD4**^**+**^ **T cell (cells/μL)**	Median (IQR)	384 (160–529)		364 (164–558)		N/A	
**HIV Duration (years)**	Median (IQR)	13 (4–22)		15 (6–25)		8 (4–13)^b^	
**CD4**^**+**^ **T cell (cells/mL)**	Median (IQR)	621 (396–820)		672 (422–920)		N/A	
**Time to cART Initiation**	0–12 months	121	(68)	124	(66)	2682	(23)
	> 12 months	48	(27)	48	(26)	4122	(35)^b^
	Unknown	8	(5)	16	(9)	4964	(42)
**Duration on cART (years)**	Median (IQR)	11 (3–18)^a^		11 (5–21)		6 (3–10)^b^	
**Ever Diagnosed with AIDS-defining Illness**		41	(23)	46	(24)	2208	(19)
**On cART **		175	(99)	184	(98)	10190	(99)
**On cART and undetectable viremia**		162	(93)	176	(96)	8889	(87)
**Hepatitis B (ever co-infected)**		17	(10)	18	(10)	1314	(11)
**Hepatitis C (ever co-infected)**		20	(11)	19	(10)	2644	(22)^b^
**Cytomegalovirus IgG**	Never co-infected	29	(16)	28	(15)	N/A	N/A
** **	Coinfected	144	(81)	155	(82)	N/A	N/A
** **	Unknown	4	(2)	5	(3)	N/A	N/A
**Ever Diagnosed with Cancer**		15	(8)	17	(9)	380	(3)^b^

^a^Denotes *p* < 0.05 for significant difference in comparing NL (2016) vs NL (2019).

^b^Denotes *p* < 0.05 for significant difference in comparing NL (2016) vs. CANOC (2016) and associated with probability of indicated cohort more likely to have the higher value.

Timely diagnosis and cART initiation are key factors involved with preventing HIV transmission and improving the quality of life for PLWH. The median duration of HIV infection for the 2019 cohort was 15 years (IQR 6–25), with median age for starting cART being 37 years (IQR 31–46). Median baseline CD4^+^ T cell counts were 364 cells/μL of blood (IQR: 164–558) (Table 1) with two-thirds of participants (66%) initiating cART between 0–12 months of receiving their diagnosis and 26% starting at least one year post-diagnosis. This longer length of time to cART initiation and prevalence of acquired immune deficiency syndrome (AIDS)-related illnesses for one quarter of the participants observed is consistent with the older age and longer duration of HIV infection within the NL HIV cohort (Table 1).

To determine the effectiveness of cART, we evaluated CD4^+^ T cell counts and viral load levels. The median CD4^+^ T cell counts for the 2019 NL cohort were 672 cells/mL (IQR: 422–920) (Table 1). Most participants (n = 176; 96%) were on cART with undetectable viremia (Table 1). The proportion of participants with detectable levels of HIV were noted to have either recently started or switched cART regimes based on dates of ART initiation, or viral load levels were measured just above the cutoff limits of detection.

### Quality of care in the NL HIV clinic from 2016 to 2019

Comparing data collected from the 2019 participants with the cohort attending clinic in 2016, identified 140 persons in common between the 2016 and 2019 cohort–an approximate 25% change from 2016. Thirty-two participants were lost between 2016 and 2019 –nine were deceased, 17 moved out of province, 2 were followed less frequently at patient request, and 4 were lost to follow up care. Of the 177 active in care in 2016, there was no significant difference in median age, gender, race, age at which participants first received cART, CD4^+^ T cell counts or duration of infection (Table 1). Further, there was no change for the time in which it took for a participant to be linked to care or those prescribed and on cART with undetectable viral loads (Table 1).

### Contrasting care in NL with Canadian clinics

The results of comparing demographic and cascade of care characteristics from the 2016 NL cohort (n = 177) with CANOC data from 2016 (n = 11 768), are described in Table 1. The median age of participants in the NL HIV clinic was greater than that of CANOC participants (OR [95%CI]: 1.02 [1.00, 1.03], *p* = 0.004), yet the age at which participants first started cART was lower for NL HIV clinic than CANOC (OR [95%CI]: 0.98 [0.97, 1.00], *p* = 0.044) (Table 1). Participants attending the NL clinic have lived longer with HIV (OR [95%CI]: 1.10 [1.08, 1.12], *p* < 0.001) than PLWH in CANOC, were on cART for a greater length of time (OR [95%CI]: 1.19 [1.15, 1.23], *p* < 0.001) and started cART earlier than CANOC participants (OR [95%CI]: 0.24 [0.17, 0.33], *p* < 0.001) (Table 1). The prevalence of HCV coinfection was less than the national cohort (OR [95%CI]: 0.43 [0.27, 0.68], *p* < 0.001); however, cancer diagnoses were more common within the NL HIV cohort (OR [95%CI]: 2.77 [1.62, 4.76], *p* < 0.001) (Table 1).

### High engagement in care and virological outcomes in PLWH in NL

For this retrospective study, it was assumed that everyone diagnosed as living with HIV was included in our NL cohort. All participants included herein are included in the first two stages of the care cascade: (1) diagnosed with HIV and (2) are linked to HIV care. If we consider participants anticipated to attend the NL clinic in 2016, as determined by 2015 data, 13 of these participants did not have a recorded visit for the 2016 calendar year. Therefore, the (3) retention in care rate for the NL cohort is 93% (177/190), not considering deaths or interprovincial moves. To assess whether the NL Provincial HIV Clinic was on par to meet the last two ‘95’ goals, we ascertained (4) the proportion of participants on cART, and (5) the number of those on cART achieving viral suppression.

**Fig 1:**
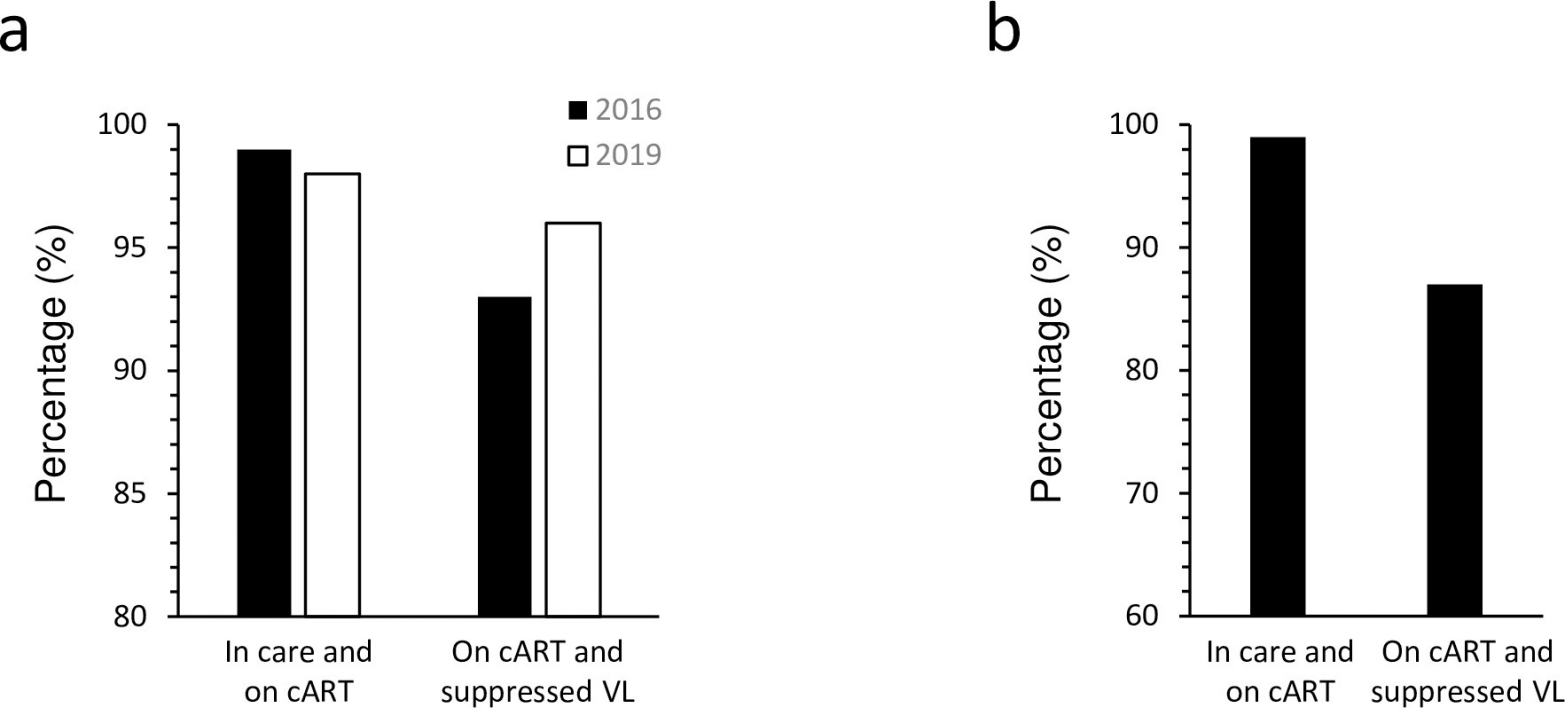
The HIV care continuum in NL and CANOC. Percentages of participants engaged in care and on cART (cascade 4) and those on cART with a suppressed VL (cascade 5) are depicted for (a) the 2016 (black bar) and 2019 NL cohorts (white bar) and (b) CANOC (black bar).

Nearly all active NL HIV clinic attendees received and were taking cART in 2016 (n = 175; 99%) and 2019 (n = 184; 98%), of which 93% of the 2016 participants (n = 162) were virologically suppressed, with this fraction rising to 96% being virologically suppressed in the 2019 cohort (n = 176) (Fig 1A). In comparison, 99% (n = 10 190) of the participants actively retained in CANOC care (n = 10 333) were on cART and of those, 87% (n = 8 889) were virologically suppressed (Fig 1B).

## Discussion

To assess the quality of care provided to PLWH by the Provincial HIV clinic in NL, we retrospectively summarized general demographics of our cohort, measured retention in care, cART use and virologic response. Delayed cART initiation is a challenge to the 95-95-95 targets as untreated HIV leads to poor individual health outcomes and can contribute to community HIV burden. Although most participants in NL started antiretroviral therapy within their first year of diagnosis, 26% of PLWH did not start cART until more than 12 months after diagnosis. This longer length of time to cART initiation and higher prevalence of acquired immune deficiency syndrome (AIDS)-related illnesses for one quarter of the participants reflect the age and duration of HIV infection within the NL HIV cohort. Given the high proportion of patients in NL who have been living with HIV for decades, this likely reflects changes in treatment guidelines over time. These guidelines ranged from recommendations to delay treatment until symptomatic when only single or dual therapy regimens were available, to starting treatment at various CD4^+^ T cell count thresholds after cART became available (to balance efficacy with long-term toxicity of treatment), to now recommending treatment as soon as possible following diagnosis (reflecting improved safety of antiretroviral therapies).

Guidelines for initiating treatment evolved with increased resources and better evidence for the benefits of early treatment. In the early 2000s, WHO recommended treatment be started when CD4^+^ T cell levels were at or below 200 cells/mm^3^ [16]. This threshold increased in 2010 and again in 2013 (350 cells/mm^3^ and 500 cells/mm^3^, respectively) with recommendations now encouraging those with new diagnoses to start cART as soon as possible, where resources permit, regardless of clinical stage and CD4^+^ T cell counts [16, 17]. New HIV diagnosis for a large proportion of the NL cohort occurred during a time in which treatment initiation could have been delayed based on WHO-recommended CD4^+^ T cell counts. Others who experienced a delayed start to cART were diagnosed outside of Canada where access to cART may not have been available.

From 2016 to 2019, most participants in the NL cohort accessed cART shortly after HIV diagnosis, were adherent and remained virally suppressed (≥ 93%) with few PLWH having detectable viremia (4–6%). In each year examined, a small fraction of viremic cases were individuals recently initiating cART after new HIV diagnosis or having made a change to their cART regime after breakthrough infection. In other cases, viremia was detected just over the cut-off threshold definition of 200 RNA copies/mL of blood, or the participant was experiencing a transient episode of detectable viremia (blip). Although there was considerable overlap in participants between 2016 and 2019, it is worthy to note that the 75% of persons that do overlap are consistent in their treatment, and the 25% new additions between 2016 and 2019 also received a high standard of care. Although the NL HIV cohort exceeds current national goals of 90% receiving cART and 90% of those persons achieving undetectable VL, there is room for improvement in order to meet the UNAIDS 95-95-95 goals by 2025. New infections continue to occur, indicating a need for enhanced surveillance methods to prevent new infections and enhance timely linkage to care.

Adherence to cART can reflect how well an individual is engaged and retained in clinical care. Despite its rural challenges, the provincial HIV clinic based in St. John’s, NL retains a high proportion of its patients. Compared with other clinics in cities servicing larger populations of PLWH, NL has relatively low cases of HIV and follow up care can be more easily monitored on an individual basis. Retention in care is correlated with improved virologic outcomes, though continuous retention may be a more meaningful predictor than cross-sectional measures of retention in care, as in our study [18]. As every HIV clinic experiences loss of participants, either due to moving out of province or country, death, or lost to care, it is necessary to implement a means to accurately identify and support those disengaged from care as well as those who are at risk of being lost to follow up care. At the start of the 2020 SARS-CoV-2 pandemic in NL, in-person clinic visits were replaced with virtual meetings and biannual labs tests and measures for virus load were significantly limited or delayed by public health restrictions. Further, testing rates for HIV declined rapidly during this time [19]. It will be prudent to contrast the 2019 clinical data presented here to that gathered during the height of stay-at-home measures imposed for the SARS-CoV-2 pandemic. Augmented efforts to identify new HIV diagnosis in NL and link these individuals with clinical services will ascertain the impact of reduced testing in terms of HIV incidence and whether there were significant changes to the progress the NL clinic made towards achieving UNAIDS 95-95-95 in previous years.

## Study limitations

As this study uses a retrospective design, it relies on accurate record-keeping. Since the NL clinic provides care to all patients with HIV infection in the province, we assumed that we are not missing patients who have been diagnosed but are not linked with care. Our clinic numbers closely align with provincial numbers for new HIV diagnoses, which were quite low (e.g. 4–8 cases/year) and we have close reporting with provincial public health teams; however, we are unable to confirm this with certainty. We reported that the NL demographic had a higher median age than CANOC with NL participants living with HIV longer and being prescribed cART for a greater length of time than CANOC participants. These differences could be attributed to the distinct inclusion criteria for each cohort, where the NL cohort includes patients diagnosed and started on treatment long before 2000, which was the entry point for the CANOC cohort. With relatively few cases of HIV in NL, the sample size is small and as such PLWH diagnosed in any year were included in this analysis whereas CANOC only considers treatment-naïve PLWH who started cART after 2000. To exclude anyone in the NL cohort who started cART before 2000 would have reduced our cohort size by approximately half. Further, the inclusion criteria for the NL cohort precluded any participants who were anticipated to attend clinic based on data from the preceding year. Efforts are being made to account for persons who are more prone to be lost to follow-up care or to contact those who have been lost to care. For these reasons, comparisons between the NL clinic and CANOC may be skewed, and statistical comparisons between the cascade of care variables for the NL cohort and CANOC were not drawn.

## Conclusions

Engagement in care and virologic outcomes for PLWH receiving care through the NL Provincial HIV program is high and compares favorably to a national cohort. Future study should assess the impact of SARS-CoV-2 pandemic on quality of care to ensure PLWH continue to achieve optimal health outcomes.
